# Editorial

**DOI:** 10.1120/jacmp.v3i4.2549

**Published:** 2002-09-01

This issue closes out Volume 3 of the JACMP. I am pleased to note that the journal continues to grow in size and outreach. Volume 2 was 60% larger (both in the number of manuscripts and the number of pages published) than Volume 1 and Volume 3 is 60% larger than Volume 2. In three years the journal has more than doubled in size.

The monthly usage statistics for the journal have also shown a steady increase, both in requests and in the unique hosts accessing the journal, during these three years.

Of particular interest has been the number of different countries from which manuscripts have been submitted. In addition to the United States and Canada, papers have come from fourteen others countries. Although the majority of the manuscripts come from the United States the percentage of submissions from other countries has increased in the last few months. Because an electronic journal is accessible worldwide it has always been editorial policy to serve the international medical physics community, and it is gratifying to see this taking place.

The success of the journal and its growth would not have been possible without the continued support of the American College of Medical Physics, who along with the journal's sponsors and advertisers have continued to underwrite the cost of the journal. This means that in 2003 Volume 4 will again be subscription free and available to all medical physicists.

The completion of Volume 3 also marks the end of my tenure as Editor‐in‐Chief and that of Georgeanne Moore as Manuscript Manager. Much of the success of the journal has been due to her hard work and handling of the daily mechanics of running the journal. The journal is very fortunate that Dr. Edwin McCullough will be taking over as Editor‐in‐Chief and Sarah Evers as the Manuscript Manager. We wish them well and look forward to continued growth and success of the journal in their capable hands.

Peter R. Almond, Ph.D.

Editor‐in‐Chief

